# Effect of In Situ NbC-Cr_7_C_3_@graphene/Fe Nanocomposite Inoculant Modification and Refinement on the Microstructure and Properties of W18Cr4V High-Speed Steel

**DOI:** 10.3390/ma17050976

**Published:** 2024-02-20

**Authors:** Lina Bai, Guixing Zheng, Lijie Zhang, Shuangjin Liu, Laichun Xu, Haowen Zheng, Jie Liu

**Affiliations:** 1 Key Laboratory for New Type of Functional Materials in Hebei Province, School of Materials Science and Technology, Hebei University of Technology, Tianjin 300130, China; 2 Department of Basic, Army Military Transportation University, Tianjin 300161, China; jsj_tj@126.com (G.Z.); 18920810661@163.com (L.Z.); lcxu11@163.com (L.X.); jiejiechonger@163.com (J.L.); 3 Department of Automation and Intelligent Science, College of Artificial Intelligence, Nankai University, Tianjin 300350, China; zhw142857@163.com

**Keywords:** W18Cr4V high-speed steel, nanocrystalline inoculant, mechanical properties, grain refining

## Abstract

A novel graphene-coated nanocrystalline ceramic particle, iron-based composite inoculant was developed in this study to optimize the as-cast microstructure and mechanical properties of W18Cr4V high-speed steel (HSS). The effects of the composite inoculant on the microstructure, crystal structure, and mechanical properties of HSS were analyzed using transmission electron microscopy, scanning electron microscopy, energy dispersive spectroscopy, and X-ray diffraction. The (00$\overset{-}{2}$) and (020) crystal planes of the Fe_3_C and Cr_7_C_3_ phases, respectively, were collinear at two points in the reciprocal space, indicating a coherent relationship between the Fe_3_C and Cr_7_C_3_ phases in the tempered modified HSS. This contributed to an improved non-uniform nucleation rate and refining of the HSS grains. The mechanical properties of the modified steel exhibited a general improvement. Specifically, the modification treatment enhanced the hardness of HSS from HRC 63.2 to 66.4 and the impact toughness by 48.3%.

## 1. Introduction

As the most common tungsten high-speed steel (HSS) grade, known as T1 HSS in the United States and SKH2 HSS in Japan, W18Cr4V plays a key role in industrial production. W18Cr4V belongs to the ledeburitic steel, which has high hardness and wear resistance and is one of the important tool steels. Specifically, W18Cr4V is more affordable than super-hard materials, making it the most suitable material for cutting tools and drills in various machines. Moreover, it is employed in high-temperature bearings that are required in the aviation field, highly temperature-resistant rolls, and high-load molds [1,2]. The microstructure of as-cast W18Cr4V mainly comprises δ eutectoid, martensite, residual austenite, and eutectic martensite [3,4]. The stability of the microstructure and mechanical properties of HSS directly ensure the safety and efficiency of mechanical equipment made from HSS. Current microalloying technologies primarily focus on the effects of different alloying and microalloying elements on steel materials [5,6,7]. However, W18Cr4V is a high-alloy steel, and incorporating excessive alloying elements can lead to compositional segregation, which is difficult to resolve. Li et al. reported that a modification in GCr15 steel increased impact toughness by 32.02% [8]. Further, Belyanchikov et al. reported that nitrides of Nb, V, Zr, and other elements promoted the precipitation of MC type carbides in the molten HSS [9].

In this study, we developed a novel nanocrystalline composite inoculant to address the severe component segregation problem in W18Cr4V. The preparation cost of the new compound inoculant is lower than that of powder metallurgy HSS. Moreover, it does not alter the cold and hot processing of HSS ingots and is suitable for large-scale industrial production [10,11,12,13]. We also investigated the microstructure of W18Cr4V after modification with the new nanocomposite inoculant to study its effects on the mechanical properties of HSS.

## 2. Experimental Procedure

### 2.1. Preparation of Composite Inoculants

Based on the thermodynamics and mismatch theory of metal materials, graphene packages of NbC-Cr_7_C_3_@graphene/Fe were prepared via in situ reaction and mechanical grinding (GN-2, Simoloyer Mill, Oute Vacuum Technology Co., Ltd., Shenyang, China) [14]. The in situ reaction ceramic particles were prepared using a vacuum tube furnace (SK-G06143, Zhonghuan Electric Furnace Co., Ltd., Tianjin, China). The process of preparing Cr_7_C_3_ and NbC in situ reaction ceramic particles was as follows: chromium powder and graphene were mixed at a mass ratio of 12:1, heated in the vacuum tube furnace to 1160 °C, held at this temperature for 0.5 h, and then cooled in the furnace. Niobium powder and graphene were mixed at a mass ratio of 10:1, heated in the vacuum tube furnace to 1200 °C, held at this temperature for 1 h, and then cooled in the furnace.

The composite inoculants were processed by wet milling using a GN-2 Simoloyer mill at a speed of 700 rmin^−1^ for mechanical grinding. The inner wall size of the ball milling jar is ϕ60 × 100 mm and the material is GCr15 bearing steel. Tungsten steel balls with diameters of 2 mm, 5 mm, and 8 mm were added to the ball milling jar, and the corresponding mass ratio of the three kinds of grinding balls was 1:2:5. The high-energy ball milling method employs the rotation or vibration of a ball mill to create a strong impact, grinding, and stirring action on the raw materials. This process effectively transfers kinetic energy to the target material through a stirrer, enhancing energy utilization and material performance. Consequently, it has become an essential method for the preparation of nanomaterials.

### 2.2. Casting and Performance Testing

W18Cr4V HSS was melted and inoculated in a vacuum induction furnace (ZG-0.025, Alkaline Vacuum Furnace Co., Ltd., Jinzhou, China) with a vacuum degree of 6.67 × 10^−3^ Pa. W18Cr4V was completely melted, inoculated with 0.5 wt.% composite inoculant, electromagnetically stirred for 2 to 3 min, and poured into a ϕ70 mm cast iron mold. The W18Cr4V cast and tempered samples were cut into cubes of side length 10 mm. Before observing the microstructure, one surface of the samples was polished with sandpaper, then mechanically polished, and a 4% dilute nitric acid solution was used for corrosion.

The mechanical properties of multiple control test steels, such as hardness, wear resistance, and impact toughness, were also examined. W18Cr4V is commonly used in high-temperature applications. Therefore, it must maintain high hardness at high temperatures. Hardness testing (HR-150A, Rockwell, Dianying Optical Instrument Co., Ltd., Shanghai, China) requires that no fewer than five points on each stress surface of the sample be tested; the overall number of measurements should not be less than 20. The red hardness test involves heating the samples in a box resistance furnace to 600 °C, maintaining them at this temperature for 60 min, and removing them for air cooling; the process must be repeated four times. Impact toughness characterizes the ability of HSS to absorb impact energy when subjected to an impact load. The experiment was conducted using a pendulum impact-testing machine (JB-200, Zhongke Metrology Instrument Co., Ltd., Yangzhou, China) at room temperature. The notchless test samples were processed to dimensions of 10 × 10 × 55 mm. A wear resistance test was conducted using a wear tester (M-200, AVIC Era Instrument Equipment Co., Ltd., Beijing, China). In the comparative experiment, each group consisted of 15 samples. The stains on the surface of the samples (10 × 10 × 12 mm) were first cleaned with acetone solution. The samples were worn for 90 min at 200 N.

Further, the properties of the new inoculant and its effects on the as-cast and tempered structures of W18Cr4V HSS were systematically studied. The influence of nanocrystalline ceramic particles present in the inoculant on the carbides in the steel and the refinement mechanism were evaluated using metallographic microscopy (Axio Imager M2m, Carl Zeiss., Oberkochen, Germany), scanning electron microscopy (SEM; Hitachi S-4800, Hitachi., Tokyo, Japan), high-resolution transmission electron microscopy (HRTEM; Jeol 2011, JEOL., Ltd., Tokyo, Japan), energy spectrum analysis, X-ray diffraction (XRD; Bruker D8 FOCUS, Brooke Co., Ltd., Saarbrucken, Germany), and Digital Micrograph 3.2 software (AMETEK, Inc., Berwyn, IL, USA).

## 3. Results and Discussion

### 3.1. Detection and Analysis of the Nanocomposite Inoculant

**Figure 1:**
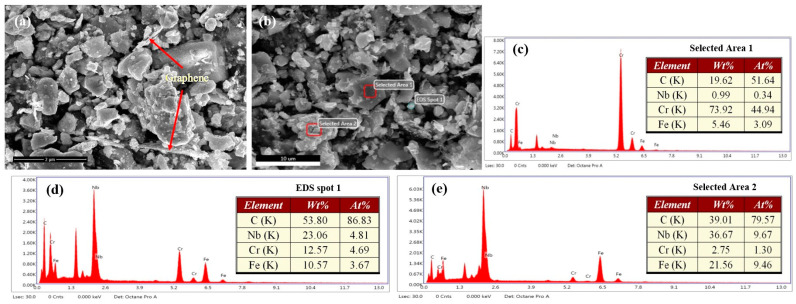
(**a**) SEM image of the composite power before milling; (**b**) SEM micrographs of the composite inoculant for 4 h of ball milling; (**c**) EDS spectra of Area 1 in Figure 1b; (**d**) EDS spectra of Spot 1 in Figure 1b; (**e**) EDS spectra of Area 2 in Figure 1b.

The SEM results revealed that the powder mixture comprised NbC ceramic particles, Cr_7_C_3_ ceramic particles, cast iron powder, and graphene at a mass ratio of 5:5:4.5:0.5 (Figure 1a). Furthermore, flake graphene was observed to be mixed in the metal particle gaps. Figure 1b shows the SEM image obtained from high-energy ball milling of the inoculant raw material for 4 h. The microstructure of the composite inoculant comprised numerous small dark gray, light gray, and bright white particles, along with a small amount of larger particles, some of which had bright white edges. Figure 1c shows the energy spectrum of Area 1 in Figure 1b, indicating the percentages of C, Cr, Nb, and Fe in Area 1 as 51.64 at.%, 44.94 at.%, 0.34 at.%, and 3.09 at.%, respectively. The energy spectrum results for Area 1 indicated that the dark gray particles were mainly chromium carbide; using the XRD analysis results, they were determined to be Cr_7_C_3_. Figure 1d,e show the energy spectra of selected areas, Spot 1 and Area 2, respectively, from Figure 1b. The bright, white-selected areas of Spot 1 and Area 2 had significantly higher C content, reaching 86.83 at.% and 79.57 at.%, respectively. Moreover, the C content in these selected areas was higher than that required to form carbides, indicating that the excess C in the composite inoculant was graphene, which formed a coating on the surface of the ceramic particles.

**Figure 2:**
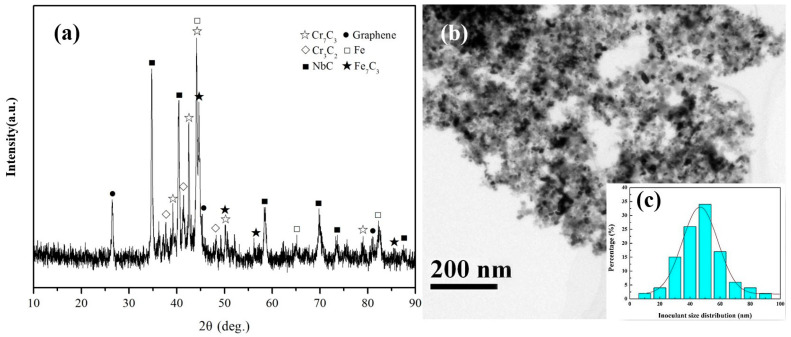
(**a**) XRD patterns of the composite inoculant for 10 h of ball milling; (**b**) TEM image of the inoculant for 10 h of ball milling; (**c**) the inoculant size distribution in Figure 2b.

The XRD pattern of the composite inoculant after 10 h of high-energy ball milling is shown in Figure 2a. The main phases in the inoculant were Cr_7_C_3_, Fe (ferrite), Fe_7_C_3_, NbC, and graphene. A small amount of the Fe_7_C_3_ phase was found in the composite inoculant, indicating that prolonged high-energy ball milling increased the atomic potential energy, resulting in Fe replacing a small amount of Cr in the iron powder. Figure 2b shows a TEM image of the NbC-Cr_7_C_3_@graphene/Fe composite inoculant, revealing that the composite inoculant particles were significantly refined through long-term high-speed rotation and collision with the steel balls. The average composite inoculant grain size (Figure 2b), measured using Image-Pro software (Figure 2c), was less than 50 nm, with a clear agglomeration of the nanocrystals. Ball milling for 10 h enables the composite inoculant to achieve nanocrystalline morphology that meets the experimental requirements, compared with ball milling for 4 h.

**Figure 3:**
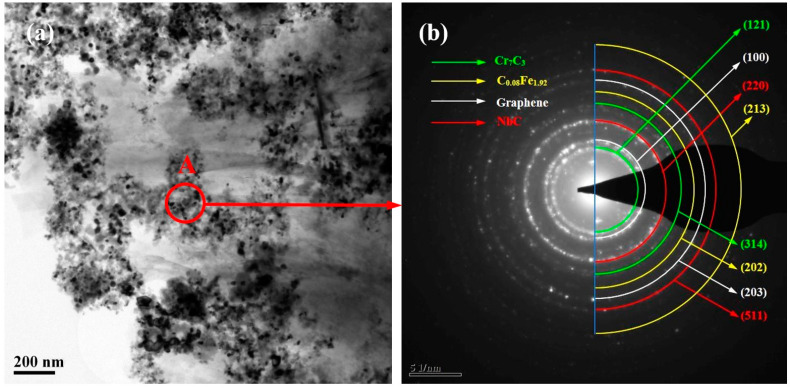
(**a**) TEM low image of the NbC-Cr_7_C_3_@Graphene/Fe inoculant for 10 h of ball milling; (**b**) SAED pattern of region A in Figure 3a.

Figure 3a shows a TEM image of the NbC-Cr_7_C_3_@graphene/Fe composite inoculant after 10 h of high-energy ball milling, revealing a significant refinement of the inoculant particles, which resulted in the ceramic particles reaching nanocrystal size. The polycrystalline diffraction ring in Figure 3b reveals the SAED pattern of region A in Figure 3a. The main phases in region A, determined through Digital Micrograph software measurement and PDF card analysis, include the NbC, Cr_7_C_3_, graphene, and C_0.08_Fe_1.92_ phases.

**Figure 4:**
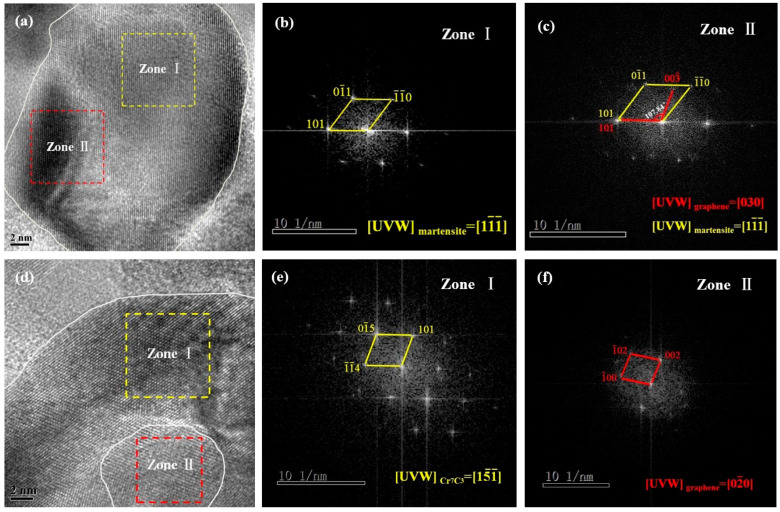
(**a**) HRTEM image of the inoculant; (**b**) FFT of Zone I in Figure 4a; (**c**) FFT of Zone II in Figure 4a; (**d**) HRTEM image of the inoculant; (**e**) FFT of Zone I in Figure 4d; (**f**) FFT of Zone II in Figure 4d.

To further determine the microstructure and phase composition of the composite inoculant using HRTEM, fast Fourier transform (FFT) analysis was performed on Zones I and II of the images (Figure 4a). Digital Micrograph software was used to measure the crystal plane spacing (Figure 4b); they were found to be 2.12 Å and 2.05 Å, with an angle of 122.36° between crystal planes. According to the calculations and PDF cards, the crystal planes in Zone I were (101) and ($\overset{-}{1}\overset{-}{1}0$), and the included angle between the crystal planes was 120°. Thus, the phase in Zone I was determined to be C_0.12_Fe_1.88_ (martensite) through a combination of formula calculations and software measurements. Two sets of reciprocal lattices appear in the FFT of Zone II, as shown in Figure 4c. Zone II was assumed to comprise graphene and martensite via analysis and calculation, indicating that the lamellar structure of graphene was torn during high-energy ball milling. Moreover, the single-layer graphene tightly adhered to the surface of ceramic particles under the strong impact of the grinding ball and ceramic particles, resulting in an overlap of the two phases in Zone II. Furthermore, the orientation difference between the crystal planes of the two phases was 14°31′. The particle phases of the composite inoculant were partitioned in the HRTEM image (Figure 4d), and FFT analysis was performed in the selected area. The FFT diffraction patterns of Zones I and II are shown in Figure 4e,f, respectively. The software analysis and calculation results indicated that Zone I comprised the Cr_7_C_3_ phase, whereas Zone II comprised the graphene phase. Thus, the experimental results revealed that layered graphene had a coating effect on Cr_7_C_3_ ceramic particles.

### 3.2. Influence of the Nanocomposite Inoculant on the Microstructure of W18Cr4V HSS

**Figure 5:**
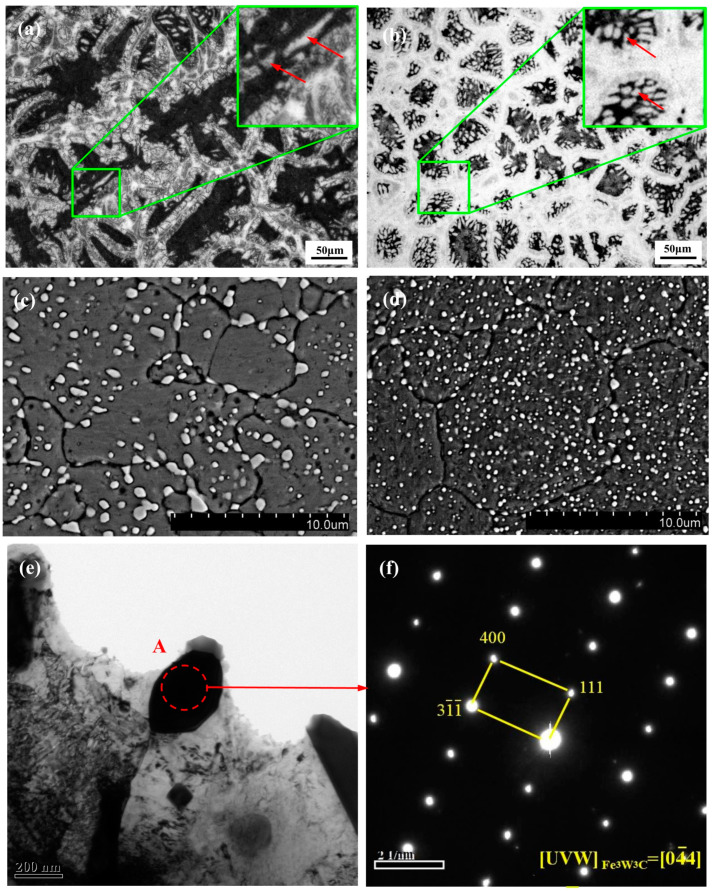
Microstructure of the as-cast HSS: (**a**) optical micrographs of the unmodified HSS; (**b**) optical micrographs of the modified HSS. SEM images of the tempered HSS in the states of (**c**) microstructure of the unmodified HSS; (**d**) microstructure of the modified HSS. TEM images of the tempered modified HSS: (**e**) TEM lower image of the tempered modified HSS; (**f**) SADE pattern of region A in Figure 5e.

Metallographic microscopy results reveal that the as-cast microstructure of W18Cr4V comprises fishbone-like eutectic carbides, a black eutectoid at the grain center, and bright white residual austenite distributed along the grain boundaries (Figure 5a,b). The as-cast microstructure of the modified W18Cr4V was significantly refined; the continuous distribution of fishbone-like eutectic carbides was reduced and disconnected, reducing the effect of splitting on the steel matrix, network carbide thickness, and grain size. In the enlarged image of the area (Figure 5a), intragranular carbides appear as strips and are unevenly distributed. However, the enlarged area in Figure 5b shows granular and more uniform intragranular carbides. Furthermore, the amount and size of fishbone carbides distributed along the grain boundaries in the as-cast modified structure were significantly reduced; the distribution of carbides was relatively independent (Figure 5b). According to the Hall–Petch formula [15], the as-cast modified steel has better yield strength. Consequently, the significantly refined carbides were more likely to be crushed during forging. The tempering process for W18Cr4V is complex and requires three treatments. The temperature increase causes the alloy elements to precipitate from the steel matrix. The microstructure of the unmodified tempered W18Cr4V, observed using SEM (Figure 5c), showed coarse granular carbides mostly precipitated at the grain boundaries on the steel matrix. The modified tempered steel exhibited a change in the distribution and morphology of the carbides; the precipitated secondary carbides were smaller and more uniform (Figure 5d). The block carbides, which were distributed on the matrix and enhanced the crystal structure of HSS, were analyzed in a specific region, namely, A, to examine the particle phase (Figure 5e). Figure 5f shows the SAED image of region A. Here, the (111) plane measured using Digital Micrograph software was 6.437 Å, which is close to the calculated crystal plane spacing (6.071 Å). Furthermore, the crystal plane indices of the other two reciprocal points in the reciprocal space were calculated and compared with the reference PDF cards (#65-1347), indicating that region A was the Fe_3_W_3_C phase. The crystal band axis in this selection area was $\left[0\overset{-}{4}4\right]$ and the three crystal planes were (111), (3$\overset{-}{1}\overset{-}{1}$), and (400).

**Figure 6:**
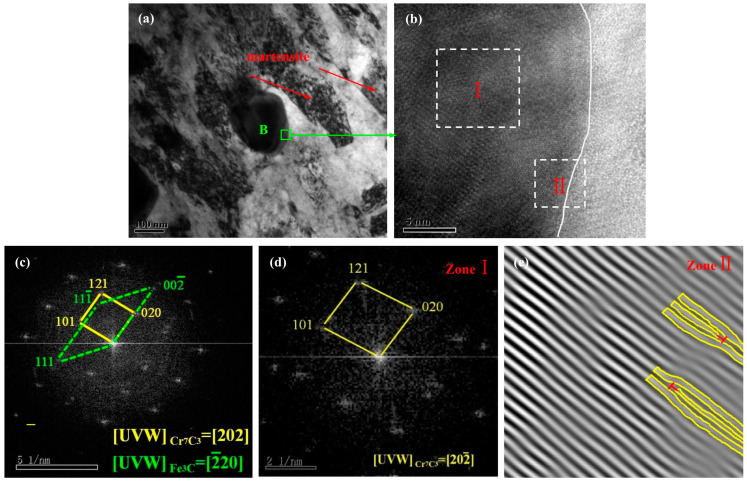
(**a**) TEM lower image showing martensitic structure and secondary carbide dispersion; (**b**) HRTEM images of region B in Figure 6a; (**c**) FFT of Figure 6b; (**d**) FFT of Zone I in Figure 6b; (**e**) inverse FFT of Zone II in Figure 6b.

Massive black carbide particles and a flat noodle martensite structure were observed on a wide range of the dark gray steel matrix in Figure 6a. The image shows that the density of carbides in steel was extremely high and the distance between carbides was less than 1 μm. When martensite makes contact with carbide in steel, its growth is hindered. This effect is similar to the fine-grained effect, because the boundary blocks the growth of martensite. Furthermore, if the grain size is less than the determined value (2–4 μm), the martensite can be dislocation structure or lath martensite [16]. Figure 6b shows an HRTEM image of region B, which is divided into two regions based on the grain boundary trend. The FFT diffraction patterns are shown in Figure 6c,d. Two sets of reciprocal lattices can be observed in the reciprocal space, as shown in Figure 6c. According to the parallelogram rule, the (00$\overset{-}{2}$) crystal plane of the Fe_3_C phase and the (020) crystal plane of the Cr_7_C_3_ phase were collinear at two points in the reciprocal space. Consequently, the (00$\overset{-}{2}$) and (020) crystal planes can be determined to be parallel and coplanar in the positive space, indicating that the Fe_3_C and Cr_7_C_3_ phases have a coherent relationship in the tempered modified HSS [17]. The FFT diffraction patterns of Zone I are shown in Figure 6d. Using software and formulas to calculate the crystal plane spacing and angle between crystal planes, Zone I was determined to comprise the Cr_7_C_3_ phase; the crystal band axis of this selected region was $\left[20\overset{-}{2}\right]$. The three crystal plane indices were (101), (020), and (121), respectively. The inverse FFT pattern of Zone II in Figure 6b is shown in Figure 6e, indicating edge dislocations at the grain boundary. These dislocations can prevent the sliding of the grain plane and improve the impact toughness of HSS. Thus, the new compound inoculant can promote heterogeneous nucleation, increase the content of primary carbides in steel, hinder the growth of martensite, and facilitate the formation of cryptocrystalline martensite.

### 3.3. Effect of the Nanocomposite Inoculant Modification on the Mechanical Properties of W18Cr4V HSS

**Figure 7:**
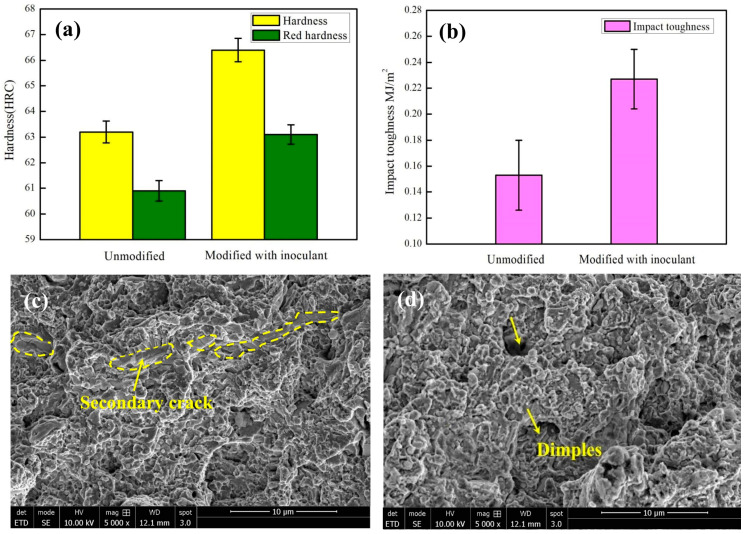
(**a**) Hardness and red hardness comparison of the tempered unmodified and modified HSS; (**b**) comparison chart of impact toughness of the unmodified and modified HSS; (**c**) SEM images of fracture morphologies of the tempered unmodified HSS; (**d**) SEM images of fracture morphologies of the tempered modified HSS.

The hardness and red hardness of the tempered W18Cr4V samples were measured and are shown in Figure 7a. The values in the graph are the arithmetic mean values of multiple measurements. The results revealed that the NbC-Cr_7_C_3_@graphene/Fe composite inoculant affected the mechanical properties of the modified HSS. The hardness of the tempered unmodified HSS was HRC (Hardness Rockwell C Scale) 63.2 and the red hardness was HRC 60.9. In addition, the hardness of the tempered modified HSS was HRC 66.4 and the red hardness was HRC 63.1. The novel composite inoculant had an impact on hardness and red hardness, resulting in a 5.1% and 3.6% increase in hardness and red hardness, respectively.

W18Cr4V is a brittle material; hence, a small pendulum (0–150 J) was used for the impact test. The results of the impact toughness tests are shown in Table 1. The impact toughness of the modified steel increased significantly from 0.153 to 0.227 MJ/m^2^ (Figure 7b). The modification treatment increased the impact toughness of W18Cr4V steel by 48.3%. The fracture morphology of the unmodified HSS was relatively shallow, as observed via SEM. Multiple continuously distributed secondary cracks were observed (Figure 7c), indicating a small bonding force between the test sample grains. The cracks propagated smoothly after being impacted by external forces, and the structure fractured along the grain plane. The morphology of the modified HSS exhibited river-like fluctuations, with many “dimples” of varying sizes; no secondary cracks were observed (Figure 7d). This indicates that the fracture of the sample occurred gradually in layers when subjected to impact force, showing increased resistance and good toughness. Owing to the refinement of the grain size of the modified HSS, the precipitation phase and impurities at the grain boundaries were also reduced [18,19]. The ceramic particle phase in the NbC-Cr_7_C_3_@graphene/Fe composite inoculant also reduced the size of the secondary carbides and increased the amount of cryptocrystalline martensite. These factors enabled the samples to absorb more deformation before fracture, resulting in better impact toughness.

**Figure 8:**
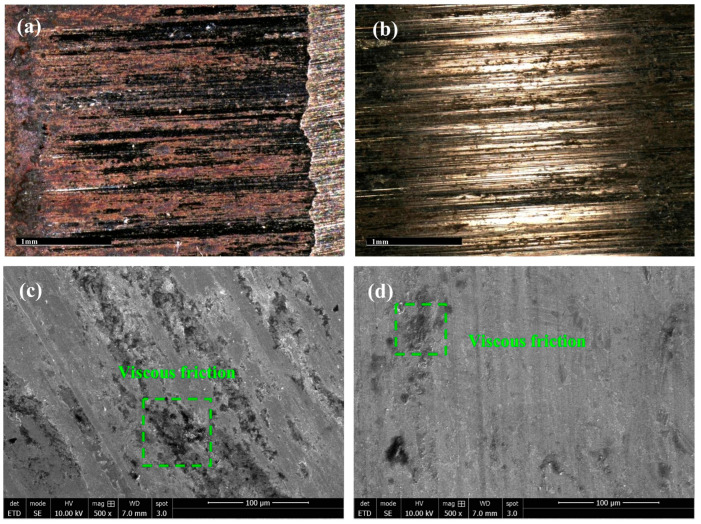
Metallographic diagram of wear surface of the tempered HSS: (**a**) unmodified HSS; (**b**) modified HSS. SEM images of wear surface of the tempered HSS in the states of (**c**) worn surface of the unmodified HSS; (**d**) worn surface of the modified HSS.

A wear resistance test was conducted using a wear tester which was worn for 90 min at 200 N. The microscopic morphology of the unmodified HSS samples exhibited deep parallel groove marks and numerous black areas on the entire surface that peeled off easily (Figure 8a). The microstructures of the modified HSS samples revealed that the groove marks on the worn surface became lighter and denser, and the black areas were significantly reduced (Figure 8b). During the friction test, adhesive wear occurred; the load applied by the testing machine to the sample caused plastic deformation on the surface of the sample. Plastic deformation causes the wear ring to come into contact with the surface of the sample under pressure, and the binding of atoms between the adhesive surfaces produces black areas. The modification treatment increased the equilibrium carbon content and carbon saturation in the steel, significantly improving the red hardness. Consequently, the black areas on the surface of the sample were reduced. During the friction test process, abrasive wear also occurred. Continuous sliding of the wear ring caused the surfaces of the samples to detach and form debris, gradually forming groove marks. However, the ceramic particles in the composite inoculant were evenly distributed in the steel matrix, improving the hardness and refining the microstructure of the steel. This resulted in shallower groove marks on the surfaces of the modified samples. The frictional wear experimental results are listed in Table 2 and show that the wear mass decreased from 10.3 to 6.1 mg. The microstructure of the unmodified steel sample was observed using SEM (Figure 8c), indicating that the martensite structure was broken, and the depth continued to increase, leading to peeling of the sample surface. However, the surface of the modified steel sample exhibited slight wear, a large light-gray area, less obvious peeling, and a lighter broken layer of the martensite structure. Moreover, dark wear pits were occasionally observed (Figure 8d).

Sexton et al. found that the wear resistance mechanism of steel materials is related to their hardness, where high hardness makes them susceptible to abrasive wear and low hardness makes them prone to adhesive wear [20]. Archard et al. derived a theory on the dependence of experiments on the load. They found that the wear rate of materials is directly proportional to the load and inversely proportional to the hardness [21]. Based on these research results, the composite inoculant not only increases the content of carbides in HSS but also improves the hardness and wear resistance of HSS, which is consistent with the experimental results of Sexton et al. During the friction and wear tests, the contact area between the test steel and wear ring increased, forming numerous dislocations and particle phases on the worn surface [22,23,24], which increased the hardness of the modified steel. The improvement in HSS red hardness resulting from modification was related to the content of carbides, with the ceramic particles coated with graphene in the composite inoculant increasing the content of alloying compounds in HSS. The alloying elements entered austenite during quenching. The higher the equilibrium carbon content and carbon saturation in steel, the higher is its quenching temperature, resulting in more carbides being incorporated into austenite. In addition, as the second-phase particle reinforcement, the high-melting-point ceramic particles in the inoculant also increased the hardness and stability of the modified steel.

### 3.4. Discussion

In the early 1970s, Bramfitt found through experimental results that TiN can reduce the nucleation undercooling of δ-Fe [25], and a two-dimensional mismatch theory was proposed based on crystal structure and data fitting. The specific formula is as follows:

(1) $$\delta_{{\left(hkl\right)}_{n}}^{{\left(hkl\right)}_{s}}=\sum_{i=1}^{3}\frac{\left|d_{{\left[uvw\right]}_{s}^{i}}\cos\theta -d_{{\left[uvw\right]}_{n}^{i}}\right|}{3d_{{\left[uvw\right]}_{s}^{i}}}\times 100\%$$

where (*hkl*)*_n_* is the low-index crystal plane of the nucleation phase, [*hkl*]*_n_* is the low-index crystal plane of the matrix phase, and [*uvw*]*_n_* and *d*_[*uvw*]*n*_ are the crystal plane index of the nucleation phase and the atomic spacing in the direction of the crystal plane index, respectively. [*uvw*]*_s_* and *d*_[*uvw*]*s*_ are the crystal plane index of the base phase and the atomic spacing in the direction of the crystal plane index, respectively, and *θ* represents the angle between these two crystal belt axes. According to the mismatch calculation formula, the planar mismatch degree of NbC and δ-Fe is 8.72% and the planar mismatch degree of Cr_7_C_3_ and δ-Fe is 6.14%. The obtained mismatch degrees were less than 12%, making it a potential heterogeneous nucleation core for the steel matrix.

**Figure 9:**
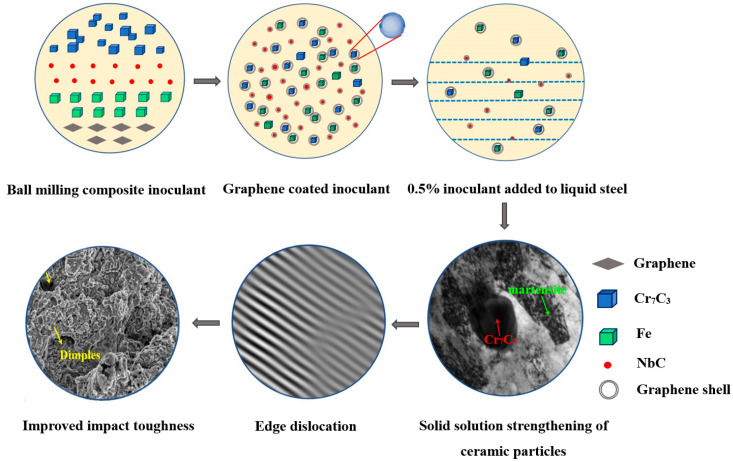
Inoculation and refinement mechanism of the NbC-Cr_7_C_3_@Graphene/Fe compound inoculants on the experimental HSS.

The NbC-Cr_7_C_3_@graphene/Fe composite inoculant was an iron-based composite inoculant, with a specific gravity very close to that of W18Cr4V metal liquid. The composite inoculants did not exhibit layering in molten steel and had a smaller wetting angle than other types of inoculants. The nanocrystalline ceramic particles in the composite inoculant were dispersed in the molten HSS, providing good nucleation support for the solidification of steel and considerably improving the non-uniform nucleation rate of the modified W18Cr4V HSS. The inoculation and refinement mechanisms of the compound inoculants in the experimental HSS are shown in Figure 9. This experiment utilized in situ reactions to prepare ceramic particle phases, mainly through precise temperature control and chemical reactions with appropriate elemental ratios. The TEM results showed that the grain size of the graphene-coated composite inoculant reached the nanoscale, and the composite inoculant had high surface energy and surface binding energy. During the fabrication process, when the composite inoculant is added to the steel liquid, the nanocrystals can easily combine with the alloy elements in the W18Cr4V steel, thereby enhancing the non-uniform nucleation rate. HRTEM analysis of the inoculant reveals that the layered structure of graphene was torn during high-energy ball milling. Under the strong collision between the grinding ball and the ceramic particles, the single-layer graphene tightly adhered to the surface of the ceramic particles, resulting in an overlap between the two phases, with a difference in crystal orientation of 14°31′. The distribution pattern of the ceramic particle phase in the HSS matrix, crystal structure, existing morphology, and the combination of the original alloying elements in the steel have a significant impact on the W18Cr4V steel. A large number of nanocrystalline ceramic particles in the composite inoculant caused more carbides to be generated during the solidification process of W18Cr4V HSS, and the grain size of the carbides was refined. NbC and Cr_7_C_3_ ceramic particles can also serve as second-phase particles to enhance intragranular strength, causing HSS to have higher hardness and better stability after modification treatment. Metallographic microscopy results revealed that the grains of the modified steel were refined, and the continuous distribution of fishbone-like eutectic carbides was disconnected. TEM results of the tempered modified HSS indicated that a large range of dark gray steel matrices were distributed with massive black Cr_7_C_3_ particles and flat noodle martensite. According to the TEM image, the density of carbides in the steel was extremely high, and the distance between carbides was less than 1 μm. The theory of the fine-grained effect indicates that Cr_7_C_3_ ceramic particles can effectively hinder the growth of carbides and martensite in steel, and an increase in alloy compounds promotes the transformation of austenite into martensite in steel, facilitating the formation of cryptocrystalline martensite. The inverse FFT pattern near the grain boundaries in HRTEM shows edge dislocations at the grain boundary. These dislocations have high strain energy, which is conducive to the precipitation of secondary carbides and can improve the mechanical properties of the modified steel. The morphology of the modified HSS exhibited a river-like fluctuation, with numerous “dimples” of varying sizes. Furthermore, no secondary cracks were observed, indicating that the fracture of the sample was gradually layered when subjected to impact force, with increased resistance and good toughness. The ceramic particle phase in the NbC-Cr_7_C_3_@graphene/Fe composite inoculant reduced the size of the secondary carbides and increased the amount of cryptocrystalline martensite. These factors enabled the samples to absorb more deformation before fracture, resulting in better impact toughness.

## 4. Conclusions

This study investigated the effect of NbC-Cr_7_C_3_@graphene/Fe composite inoculants on the microstructure and mechanical properties of W18Cr4V HSS during the modification process. The conclusions are as follows:

1. According to the XRD patterns and Jade software analysis, the main phases in the composite inoculant were Cr_7_C_3_, Fe (ferrite), Fe_7_C_3_, NbC, and graphene. The average grain size was less than 50 nm, and significant agglomeration of nanocrystals was observed in the TEM image. Furthermore, single-layer graphene tightly covered the surface of the ceramic particles under strong collisions between the grinding ball and ceramic particles, resulting in an overlap of the two phases in the selected area.
2. The as-cast microstructure of the modified W18Cr4V was significantly refined, with the continuous distribution of fishbone-like eutectic carbides reduced and disconnected. This reduction decreased the effect of splitting on the steel matrix. Furthermore, the SEM images of the tempered modified HSS indicate changes in the distribution and morphology of the carbides, showing smaller and more uniform secondary carbides.
3. The (00$\overset{-}{2}$) crystal plane of the Fe_3_C phase and the (020) crystal plane of the Cr_7_C_3_ phase were collinear at two points in the reciprocal space. This indicates that the Fe_3_C and Cr_7_C_3_ phases had a coherent relationship in the tempered modified HSS. The HRTEM results indicate that the ceramic particles provide a nucleation substrate for the carbides in W18Cr4V, improving the non-uniform nucleation rate.
4. The nanocrystalline composite inoculant had an impact on the mechanical properties of W18Cr4V. The hardness of the tempered modified HSS increased from HRC 63.2 to 66.4 and the wear mass decreased from 10.3 to 6.1 mg. Moreover, the modification treatment enhanced the impact toughness of the steel by 48.3%.

## Figures and Tables

**Table 1:** Impact energy and impact toughness of the tempered W18Cr4V HSS.

Sample Category	Impact Energy /MJ(A_k_)	Cross-Sectional Area/m^2^ (F)	Impact Toughness/(MJ/m^2^) (a_k_)
Unmodified	(1.48 ± 0.19) × 10^−5^	(9.68 ± 0.47) × 10^−5^	0.153 ± 0.027
Modified	(2.21 ± 0.23) × 10^−5^	(9.72 ± 0.36) × 10^−5^	0.227 ± 0.023

**Table 2:** Frictional wear experiments of the tempered W18Cr4V HSS.

State of HSS	Initial Mass/g	Residual Mass/g	Wear Mass/g
Uninoculated	10.3275	10.3172	0.0103
Inoculated	9.0885	9.0824	0.0061

## Data Availability

No new data were created or analyzed in this study. Data sharing is not applicable to this article.
